# Current Knowledge and Behavior towards Salt Reduction among Hong Kong Citizens: A Cross–Sectional Survey

**DOI:** 10.3390/ijerph18189572

**Published:** 2021-09-11

**Authors:** Jasmine Cheung, Deborah Neyle, Peggy Pik Kei Chow

**Affiliations:** 1 School of Nursing, Tung Wah College, Hong Kong, China; peggychow@twc.edu.hk; 2 Independent Scholar, Auburn, NSW 2144, Australia; debn2144@gmail.com

**Keywords:** dietary salt, sodium, salt reduction, salt intake, community participation

## Abstract

Excessive dietary salt intake is prevalent in the Hong Kong community. Over the last decade, the Hong Kong Special Administrative Region Government has been actively promoting community participation to reduce salt intake. The aim of this study was to investigate the current knowledge levels and behaviors relating to dietary salt intake among Hong Kong adults. This cross-sectional survey involved 426 adults in Hong Kong. The findings of this study identified areas of knowledge deficit in the recommended upper limit of daily salt intake for an adult set by World Health Organization (*n* = 295, 69.2%) indicated a lack of awareness that the overconsumption of salt could cause coronary heart disease (*n* = 233, 54.7%). Disengagement with salt reduction behavior, such as rarely/never checking the sodium or salt content listed on the food label (*n* = 252, 59.2%) and rarely/never purchasing food labelled with low salt or no salt content (*n* = 292, 68.9%), was reported. Excessive salt intake in dietary habits remains an under-recognized non-communicable disease threat by Hong Kong citizens, indicating ineffective responsive risk communication. There is a need to refine existing salt reduction initiatives to aid in making appropriate decisions regarding dietary salt intake among Hong Kong citizens.

## 1. Introduction

For at least the past decade, Hong Kong health authorities have implemented many actions directed at educating the population of Hong Kong about problems associated with excessive salt intake and at attempting to assist people to adopt behaviors that will reduce their salt intake. The goal of reducing personal salt intake by 30% by 2025 was established and is consistent with global health goals [1,2]. Salt reduction strategies have been implemented at a population level through food labelling schemes, healthy eating promotion campaigns, risk assessment measures, trade consultations, and food product reformulation [2,3,4]. However, local studies persistently show that salt intake and salt-related chronic disease are continuing to increase. Over 85% of the Hong Kong population aged 15–84 consume dietary salt above the WHO recommended daily limit [5]. According to current laboratory measurements and behavioral surveys, the mean urinary sodium excretion over 24 h among healthy Chinese adults in Hong Kong was 7.3 ± 3.2 g salt/d, which exceeds the World Health Organization standard of <5 g salt/d and the China Nutrition Society of <6 g salt/d) [5,6,7,8,9]. An increased community threat regarding excessive salt intake was reported in the headline of local newspaper: “Having double safe limit of salt as recommended by the World Health Organization”, indicating a need to review existing salt reduction initiatives and to identify knowledge and behavioral gaps at the individual level [10,11].

Salt related conditions mostly affect the cardiovascular system and manifest as hypertension, cardiac failure, and stroke [12,13]. These diseases are mostly expressed in people over 65 years of age, but in Hong Kong, nearly 10% of people aged 45–54 also suffer from hypertension, suggesting a prolonged period for the development of this condition [14]. Salt is also implicated in stomach cancer, osteoporosis, kidney disease, and autoimmune diseases [15,16,17,18,19,20,21]. The health burden in Hong Kong is demonstrated by worsening disability-adjusted life years and premature death [22].

In 2015, Chau and others developed a Chinese Health Literacy Scale for low salt consumption as a method of establishing a baseline of knowledge, attitudes, and behavior around issues of salt intake [23]. This scale covered eight areas, five of which addressed knowledge, with two addressed behaviors, and one addressed personal attitudes. The validation study for this scale was conducted on a population over 65 years of age. This age group is the most affected by the chronic disorders associated with accumulated physical insults. Low awareness of high sodium intake among older Chinese people has recently been reported [24].

The scale was developed for use in health screenings or health assessments and as a method of evaluating salt reduction interventions. It was posited as offering a much easier way of assessing salt intake than previous methods of salt assessment such as biomarkers (for instance, urinary sodium) and food diaries. However, this scale along, with other initiatives, has not been effective in changing individual behaviors. Salt is noted as a highly addictive substance [25] that affects dopamine and opiate receptors in the brain’s reward and pleasure systems [3]. This physiological attraction to salt appears to be overwhelming the educational and behavioral initiatives of the Hong Kong authorities. Three years ago, the Hong Kong government addressed the global call to shift the focus of salt reduction initiatives from consumer awareness to personal action [26]. The emphasis on knowledge levels alone may be displaced by a greater concentration on behaviors or actions adopted by an individual.

Hong Kong is not unique in this salt-related dilemma. Many countries suffer with processed food that are high in salt [27,28] as well as the personal addition of extra salt while eating. For instance, Africa reported insufficient salt reduction knowledge and behavior in rural dwellers with lower education levels [29,30]. In the Middle East, only approximately one-third of the participants are able to define processed food as a major source of salt and would purchase food with less-salt content [31]. In Greece, nearly 90% of participants did not know the exact amount of the recommended daily salt intake [32]. Comparatively, knowledge about major sources of sodium was high in Korea [33]. In Australia, while the majority of participants (90%) were aware that excessive salt intake can cause health damage, over 80% reported that they were eating “far too much” than the recommended daily intake, with less than half of the participants attempting to reduce their salt intake [34].

According to a systematic review conducted in the year 2014, populations from various countries were unable to distinguish the difference between salt and sodium or correctly identify the recommended daily salt consumption amount and the major sources of salt from diets [35]. Another review conducted across 12 high-income countries in the year of 2018 pointed out that the knowledge and behavior regarding salt reduction remains low. Fundamental knowledge regarding recommended daily intake, primary food sources of high salt content, and the differences between salt and sodium continues to be lacking [36]. The World Health Organisation goal of 2 g of sodium (5 g of salt)/day is being met in very few counties around the world [6,37]. This may be related to the addictive qualities of salt that were mentioned above. There is a lack of comprehensive analysis on the knowledge and behavior towards salt reduction in the adult population of Hong Kong.

The aim of this study was to investigate the current knowledge levels and behaviors relating to dietary salt intake among Hong Kong individuals across the full adult age range. We hypothesized that after all of the educational and behavioral initiatives around salt intake, Hong Kong adults would be knowledgeable in appropriate salt intake levels and personal salt reduction strategies. We further hypothesised that increased knowledge levels would be positively related to engagement in salt reduction behavior. While a null hypothesis could be written, we believed that the directed hypotheses were reasonable based on the plethora of educational and other initiatives in this area of knowledge and behavior in Hong Kong.

## 2. Materials and Methods

### 2.1. Variables and Measurement

A deliberate decision was made to use a well-validated research tool from Australia for this study in order to be able to link information from Hong Kong with broader global information and to reflect a greater emphasis on attitudes and behaviors. The questionnaire was adopted with the author’s permission [34]. The self-reported survey consists of 17 questions, including questions about demographic characteristics, dietary salt reduction knowledge, and actual salt consumption behavior. Minimal language modifications were made (for instance, altering Australia to Hong Kong). The content validity was rated by seven experts and achieved an acceptable value (ICVI ranging from 0.857 to 1.0 and SCVI/Ave = 0.988) [38]. Pilot studies were conducted to ensure the test–retest reliability, with the response consistency ranging from 0.817–1.0. Cronbach’s alpha was 0.739.

The study variables in this study included age, gender, education level, and advice from health professionals. Compared to other age groups, a higher mean salt intake was reported by people aged 35–44 (9.5 g/day) and people aged 45–54 (9.4 g/day) [37]. Taking into consideration that all Hong Kong adults aged 18–84 reported excessive salt intake, with their mean salt intake ranging from 7.9 g/day to 10.6 g/day, exceeding the WHO recommended daily limit of 5 g [1,5], this study set the inclusion criteria of being aged 18–84.

### 2.2. Study Design

A cross sectional study design was adopted.

### 2.3. Sampling

The inclusion criteria were set as broadly as possible and included (1) being Hong Kong citizens between 18–84 years of age, (2) having the ability to read Chinese or English, and (3) having the ability to communicate in Cantonese, English, and Mandarin. Access to participants was determined by placing eighteen names of the districts in a sealed bag. Six HK districts were then selected by simple probability sampling techniques, including Eastern, Wan Chai, Sha Tin, Tsuen Wan, Yau Tsim Mong, and Sham Shui Po. It was not predicted that there would be inter-district variation on this issue. Sample size was calculated using the formula below [39]:

n_0_ = Z^2^ × pq/e^2^ (1)

With a 95% confidence level and ±5% precision, the estimated sample size was 384.

n_0_ = (1.96)^2^ × (0.5) × (0.5)/(0.05)^2^ = 384 (2)

Considering survey dropout analysis, the total sample size = 384 × (1 + 10%) was approximately 423.

### 2.4. Data Collection

Data collection was conducted by a group of multilingual data collectors accessing members of the public in front of the district council buildings in each of the chosen districts. A total of 426 respondents were recruited in Hong Kong in February 2019.

### 2.5. Ethics Consideration

Ethics approval was granted by the School Research Committee, Tung Wah College, Hong Kong (Ref. No: NUR/SRC/20181218/006). A participant information sheet was provided to all of the respondents and informed consent was obtained from all of the participants prior to data collection. No monetary incentives were offered for completing the questionnaire.

### 2.6. Data Analysis

Data were analyzed using IBM SPSS Statistics for Windows Version 23.0 (IBM Corp, Armonk, NY, USA). Descriptive statistics including Pearson’s correlation and coefficient, chi-squared test, and ANOVA were used for data analysis, with a *p*-value < 0.05 considered to be statistically significant.

The total knowledge score and total behavior score served as the initial indicator to determine whether there was statistical significance and if further investigation was needed. The total knowledge score was calculated by assigning one mark for each of the correct answers in questions 6–10 and 11a–11d. A total 9 marks were available for the total knowledge score. The total behavior score was calculated by assigning one mark for each of the correct answers in questions 12–14 and 16. For question 15a–15f, 0–5 marks were assigned for the answers “never”, “rarely”, “sometimes”, “often usually”, and “always”, respectively. A total 34 marks were available for the total behavior score. Percentiles were used to compare and the interpret data from both the total knowledge score and the total behavior score.

We hypothesized that the middle age group would have relatively lower total knowledge scores and/or behavior scores than the other age groups. Both males and females were included in this study. While men (4.14 g/day) demonstrated a higher mean sodium intake than women (3.77 g/day) [2], both genders had high salt intake. For instance, the corresponding proportion of the consumption of snacks with high salt content was the same for both females and males. While “slightly more” females (59.4%) than males (59.1%) used salt and salted seasoning, “relatively more” males (51.3%) ate processed meat and associated products than females did (42.2%). Depending on the diet and salt-consumption behavior, both genders are at risk of the over-consumption of salt and therefore are included in this study [1]. We hypothesize that there will be gender differences in the total knowledge scores and/or total behavior scores for salt reduction. People with high education levels reported better knowledge, attitudes, and practices regarding salt intake than people with lower education levels [5]. Therefore, education level was included as one of our study variables. Seeking professional health advice is regarded as one of the supportive strategies to reduce the intake of salt [6,7]. We hypothesize that participants who have sought professional advice will have better salt reduction knowledge scores and/or total behavior scores.

## 3. Results

The profile of the respondents included the gender distribution (less than 5% difference between male and female); age (M = 36.6, SD = 13.665); education level (nearly 90% attained secondary education or above); received professional advice for reducing salt intake (33.1%) and health status (about 30% of respondents reported having chronic illness) of the participants (Table 1).

Knowledge deficit and a lack of engagement in salt reduction behavior were reported (Table 2, Table 3 and Table 4). The total knowledge score (5.98 ± 1.931 out of 9) was calculated according to correct answers given for questions 6 to 11d (9 marks) in the questionnaire. The total behavior score (14.68 ± 5.250 out of 34) was calculated based on the responses to questions 12 to 17, in which correct answers for question 12–14 and 16 would score 1 mark (4 marks), and mark 0–5 would be allocated to each answer option in question 15a–5f (30 marks).

The key findings of this study demonstrated that over half of the respondents did not know the recommended upper limit of daily salt intake for an adult set by World Health Organization (*n* = 295, 69.2%) and were not aware that the overconsumption of salt could cause coronary heart disease (*n* = 233, 54.7%) and stroke (*n* = 221, 51.9%) [4]. While most of the respondents perceived that Hong Kongers consumed an excessive amount of salt in their usual diets (*n* = 328, 77.0%), only one-third of respondents had attempted to cut down the salt intake in their daily diet (*n* = 136, 31.9%). Most of the respondents rarely or never checked the sodium or salt content listed on the food label (*n* = 252, 59.2%) or purchased food labelled with low salt or no salt content (*n* = 292, 68.9%). Less than 20% of the respondents would always/often check the low salt or no salt food label when selecting food products (*n* = 64, 19.2%). A relatively small number of respondents engaged in dietary salt reduction during home cooking, with few respondents (*n* = 81, 19.0%) often/always avoiding buying pre-packaged food or ready-to-eat food and even fewer respondents (*n* = 43, 10.1%) using spice/herbs as salt substitutes. Dietary salt reduction in dining-out behavior showed that nearly 40.0% respondents (*n* = 169) rarely/never avoided fast food, and 75.0% respondents (*n* = 318) rarely/never requested less salt in their meal. Among various sources of information regarding dietary salt reduction, people most often accessed a health professional (M = 3.84, SD = 1.011). Nevertheless, more than two-thirds of the respondents had not sought professional advice to reduce their dietary salt intake (*n* = 285, 66.9%). Respondents who had sought professional advice on salt reduction were more knowledgeable in recognizing the relationship between the overconsumption of salt and coronary heart disease (X^2^ = 6.284, df = 1, *p*= 0.012). No significance was shown between professional advice sought on dietary salt reduction and other chronic conditions, including hypertension, kidney disease and stroke. Television was the most common knowledge source used by Hong Kong citizens to acquire information regarding salt reduction (*n* = 354, 83.0%) (Table 5).

A weak positive association between the total knowledge score and the total behavior score on dietary salt reduction was shown (Pearson correlation test: r = 0.281, *p* = 0.000). Education level was the only demographic characteristic that had significant a mean difference with the total knowledge score (F(3, 422) = 6.570, *p* = 0.000) and the total behavior score (F(3, 425) = 6.422, *p* = 0.000), respectively (Table 6). A one-way between-group analysis of variance was conducted to explore the impact of education levels on the total knowledge score. Respondents were divided into four groups according to their education level (Group 1: primary or below; Group 2: secondary; Group 3: college/university; Group 4: post-graduate or above). There was a statistically significant difference at the *p* < 0.05 level in the total knowledge scores in the four education level groups F (3, 419) = 6.6, *p* = 0.00). Despite reaching statistical significance, the actual difference in the mean scores between the groups was quite small. The effect size, calculated using the eta squared, was 0.04. Post hoc comparisons using the Tukey HSD test indicated that the mean score for the primary or below group (M = 5.65, SD = 1.94) was significantly different from the postgraduate or above group (M = 7.00, SD = 1.93). The secondary group (M = 5.59, SD = 1.81) was significantly different from the college/university group (M = 6.19, SD = 1.95) and the post-graduate or above group.

## 4. Discussion

This study assessed the knowledge and behaviors related to salt reduction amongst Hong Kong citizens.

Demographic data from the study demonstrated that the average age of participants at 36.7 years in this study is comparable with the 2019 average for the Hong Kong population of 44.0 years [40]. The age distribution covers a wide range, including many individuals for whom the salt reduction initiatives of the Hong Kong government are highly relevant, that is, people within a preventative age range. The age group for whom prevention is a real option have been strongly included in this study. Gender has been evenly distributed within the study, as would be expected in a reasonable population sample.

In this survey, it appears that many Hong Kong residents do recognize the association between excessive salt intake and adverse health consequences. Consistent with the literature [35], Hong Kong citizens were able to identify health risks associated with excessive salt intake but demonstrated poor knowledge in some aspects of salt prevention, such as the recommended daily intake. The significant weak association between knowledge and salt reduction behavior may suggest that Hong Kong citizens who attempt to engage with salt reduction behavior may not benefit due to a knowledge deficit. For example, the respondents who engage in salt reduction behavior by checking the sodium and salt content listed on a food label may not be able to understand and interpret that label and therefore are not selecting foods with an appropriate sodium content. Their knowledge gap in distinguishing between salt and sodium and stating the upper limit of salt intake as recommended by the WHO are barriers for Hong Kong citizens who are willing to engage in salt reduction behavior.

The ability to personalize the association between the overconsumption of dietary salt and illness appears to be less developed. Notably, another local study reported a strong under estimation of urinary sodium levels by older people who had participated in biometric data collection [41]. This suggests that the concentration on food sodium levels has not been individualized by people, especially in their need to alter traditional food patterns.

There is a lack of engagement in reducing risky behavior towards excessive salt intake by people in Hong Kong. Compared to the 55% of respondents from a baseline survey conducted in mid-2008 in Hong Kong who reported that they “always”/“most times” read nutrition labels when they bought a food product for the first time [42], only 10.1% of the respondents checked the sodium and salt content listed on food label on each item in this study. The relatively low rate of checking the food label could be due to the respondents repeatedly purchasing the same food and being familiar with the sodium content already or may be because the respondents do not engage in food label checking behavior. Fatigue with this aspect of the public health promotion is also a possibility. In this study, only 11.8% of respondents “always”/“sometimes” purchased food labelled as “no added salt”, “salt reduced”, or “reduced sodium”. The effectiveness of food labels ought to be questioned, and the current priority of implementing the front-of-pack label ought to be questioned, and current priority of implementing front-of-pack labelling is questionable.

One priority for refining existing salt reduction initiatives would be to strengthen the existing health promotion targeting the relationship between excessive salt/sodium intake and the increased risk of stroke and cardiovascular disease. Excessive salt intake is associated with known cardiovascular disease risk factors that are increasing in prevalence in Hong Kong [43]. Excessive sodium intake as a personally modifiable lifestyle factor to reduce stroke and cardiovascular disease through advice from healthcare professionals and television-based health promotions may improve community participation [44,45].

Paradoxically, findings from this study are comparable to current literature in which the participants with higher education levels were reported to have poorer dietary habits with less consumption of low salt diets [46]. This finding is worthy of further exploration, as it strongly supports the concept that knowledge is not a significant precursor of behavioral change. Individual support and recognition of embedded social structures need to be more prominent in public health initiatives. Campaigns targeting different groups via their education levels may also foster knowledge attainment and create behavioral changes regarding dietary salt intake.

The acknowledgement of consumers as not only cognitive but also emotional beings in future research can contribute to design thinking and hence the food experiences and well-being of participants [47,48].

This study is limited in that it uses self-reporting of behaviors although the self-reporting would be expected to overestimate correct behavior. Nevertheless, the self-reporting of salt eating habits is a more effective approach to determine actual salt intake when compared to measurements of a salt to taste threshold [49]. This study did not measure urinary sodium excretion, which is in contrast to studies that have already been conducted in Hong Kong [9,50,51].

In addition to knowledge, future directions can focus on investigating health literacy, food salt awareness, and understanding dietary guidelines to inform dietary outcomes [52,53,54,55]. It is important to educate the public about scientific evidence that the salt taste detection threshold can be decreased by salt intake reduction [56,57]. Using other sources (for instance, vitamin B4) as salt substitute that do not affect the overall sensory or salty perception could be promoted to enhance consumer knowledge and to enhance their acceptance of salt reduction [58]. The findings of this study further contribute to the audience analysis part of the social marketing framework for salt reduction, in which the consumer/target audience’s current knowledge, attitudes, and behaviors related to salt consumption have been investigated and synthesized to facilitate the communication of salt reduction messages across specific cultural contexts [59]. Social marketing offers alternative options to the traditional focus of using public policies to decrease a population’s salt intake [60].

Another limitation is that an in-depth assessment of relationships between knowledge and behavior of salt reduction was limited by the nature of the cross-sectional study. Investigation of the estimated sodium intake and the expression of functional literacy and sodium appetite can enrich findings from our quantitative factors. In addition, some variables were not manipulated in this study, such as economic status and occupation. Consideration should be given to identifying potential risk factors for excessive salt intake behavior in future research, for instance, to evaluate sociocultural factors and salt intake behavior. Strengths of the study include the use of a well-validated and reliable instrument.

## 5. Conclusions

This study investigated the association between salt reduction and behavior in Hong Kong citizens. The results revealed that community participation in dietary salt reduction in Hong Kong remains a dilemma. Hong Kong has attempted to build consumer awareness towards salt reduction. However, the identified areas of knowledge deficit, the lack of awareness around the overconsumption of salt and the links to prevalent chronic conditions, demand efforts to address the lack of engagement in reducing risky behavior towards excessive salt intake. These findings can inform future action planning to refine existing salt reduction initiatives in Hong Kong and to encourage the measurement of actual behavioral changes.

## Figures and Tables

**Table 1:** Demographic characteristics (*n* = 426).

		*n*	(%)
**Mean age**			
	(years)	36.68 ± 13.665	-
**Sex**			
	Male	222	(52.1)
	Female	204	(47.9)
**Education level**			
	Primary or below	43	(10.1)
	Secondary	155	(36.4)
	College/University	195	(45.8)
	Post-graduated or above	33	(7.7)
**Sought professional advice for dietary salt reduction**			
	Yes	141	(33.1)
	No	285	(66.9)
**With one or more of the chronic health condition(s)**			
	Yes	129	(30.3)
	Hypertension	92	(21.6)
	Renal disease	23	(5.4)
	Stroke	18	(4.2)
	Coronary heart disease	22	(5.2)
	Others	10	(2.3)
	No	297	(69.7)

**Table 2:** Knowledge of dietary salt reduction (Q6–11).

	Question and Answer(s)	*n*	%
Q6.	**What is the relationship between salt and sodium?**		
	Salt contains sodium (correct)	197	46.2
	They are exactly the same	102	23.9
	Sodium contains salt	44	10.3
	Don’t know	83	19.5
Q7.	**Do you think people in Hong Kong have too much salt in the usual diet?**		
	Far too much (correct)	105	24.6
	Too much (correct)	223	52.3
	Just the right amount	59	13.8
	Too little	10	2.3
	Far too little	6	1.4
	Don’t know	23	5.4
Q8.	**Which of the following is the main source of salt in the Hong Kong diet?**		
	Salt or sauce (e.g., Soya sauce/Oyster sauce) added during cooking or at the table (correct)	184	43.2
	Salt from processed foods such as canned food, soup, breads, sausages and cheese (correct)	181	42.5
	Salt from natural food sources (e.g., celery)	24	5.6
	Don’t know	37	8.7
Q9.	**What is the recommended upper limit of daily salt intake from health professionals?**		
	5 g (about 1 teaspoon) (correct)	131	30.8
	3 g (about 1/2 teaspoon)	59	13.8
	8 g (about 1 and a 1/2 teaspoons)	57	13.4
	10 g (about 2 teaspoons)	53	12.4
	15 g (about 3 teaspoons)	22	5.2
	Don’t know	104	24.4
Q10.	**Do you think the overconsumption of salt can damage your health?**		
	Yes (correct)	399	93.7
	No	14	3.3
	Don’t know	13	3
Q11.	**Which, if any, of the following conditions is related to the overconsumption of salt?**		
	a. Coronary heart disease (correct)	193	45.3
	b. Stroke (correct)	205	48.1
	c. Renal disease (correct)	346	81.2
	d. Hypertension (correct)	365	85.7

**Table 3:** Dietary salt reduction behavior (Q12–14, 16).

Question and Answer(s)		*n*	%
Q12.	**How often do you add extra salt or salty sauce (e.g., Soya sauce/Oyster sauce) to your meal at meal time?**		
	Rarely (correct)	143	3.1
	Never (correct)	13	33.6
	Always	41	9.6
	Often	85	20.0
	Sometimes	142	33.3
	Don’t know	2	0.5
Q13.	**How often do you add salt or salty sauce (e.g., Soya sauce/Oyster sauce) to season food while cooking at home?**		
	Rarely (correct)	98	23.0
	Never (correct)	14	3.30
	Always	72	16.9
	Often	121	28.4
	Sometimes	119	27.9
	Don’t know	2	0.5
Q14.	**Have you tried to cut down the salt intake in your daily diet?**		
	Yes (correct)	136	31.9
	No	263	61.7
	Don’t know	27	6.3
Q16.	**Would you pay attention to a “Low salt” or “No salt” food label during shopping?**		
	**Always (correct)**	19	4.5
	**Often (correct)**	31	7.3
	**Sometimes**	82	19.2
	**Rarely**	137	32.2
	**Never**	155	36.4
	**Don’t know**	2	0.5

**Table 4:** Dietary salty reduction behavior(Q15).

How Often Have You Applied the Following Salt Reduction Strategy(ies) in the Past One Month?	Always (%)	Often (%)	Sometimes (%)	Rarely (%)	Never (%)	Do Not Know (%)
a. Check the sodium and salt content listed on the food label on each item	2.6	7.5	29.7	34.4	25.7	0
b. Avoid eating pre-packaged or ready to eat foods	3.3	15.8	33.6	31.2	16.1	0
c. Use spices/herbs as a substitute for salt when cooking	2.4	7.8	21.3	31.9	36.6	0
d. Avoid eating food from fast food restaurants	5.0	20.1	35.0	28.6	11.3	0
e. Purchased food labelled “no added salt”, “salt reduced”, or “reduced sodium”	4.5	7.3	19.3	32.3	36.6	0
f. Request a salt-free diet or a low salt diet when dining out	2.1	4.2	18.2	23.3	51.7	0

**Table 5:** Five of the most common information sources regarding salt reduction used by Hong Kong citizens (*n* = 426).

	*n*	%
Pamphlet/poster	315	73.9
Newspaper/magazine	323	75.8
Television	354	83.1
Health professionals	307	72.1
Friend/family/parent	324	76.1

**Table 6:** Education level and total knowledge score/total behavioral score (ANOVA).

	F	Sig.
Education level and Total knowledge score	6.570	0.000 *
Education level and Total behavior score	6.422	0.000 *

Notes: (1) Abbreviations: ANOVA, analysis of variance; F, ratio of two sample variance; (2) * statistical significance (*p* < 0.05).

## Data Availability

The data presented in this study are available upon request from the corresponding author.
